# Molecular Marker Identification for Relapse Prediction in 5-FU-Based Adjuvant Chemotherapy in Gastric and Colorectal Cancers

**DOI:** 10.1371/journal.pone.0043236

**Published:** 2012-08-14

**Authors:** Kazushige Ishida, Satoshi S. Nishizuka, Takehiro Chiba, Miyuki Ikeda, Kohei Kume, Fumitaka Endo, Hirokatsu Katagiri, Teppei Matsuo, Hironobu Noda, Takeshi Iwaya, Noriyuki Yamada, Hisataka Fujiwara, Masanori Takahashi, Tetsuya Itabashi, Noriyuki Uesugi, Chihaya Maesawa, Gen Tamura, Tamotsu Sugai, Koki Otsuka, Keisuke Koeda, Go Wakabayashi

**Affiliations:** 1 Department of Surgery, Iwate Medical University School of Medicine, Morioka, Japan; 2 MIAST (Medical Innovation by Advanced Science and Technology), Iwate Medical University, Morioka, Japan; 3 Department of Surgery, Medical Institute of Bioregulation, Kyushu University, Beppu, Japan; 4 Department of Tumor Biology, Center for Advanced Medical Science, Iwate Medical University, Yahaba, Japan; 5 Division of Diagnostic Molecular Pathology, Department of Pathology, Iwate Medical University School of Medicine, Morioka, Japan; 6 Department of Pathology and Laboratory Medicine, Yamagata Prefectural Central Hospital, Yamagata, Japan; The Chinese University of Hong Kong, Hong Kong

## Abstract

To confirm the clinical significance of NF-κB and JNK protein expression from experimentally identified candidates for predicting prognosis for patients with 5-FU treatment, we evaluated the protein expression of surgically removed specimens. A total of 79 specimens were obtained from 30 gastric and 49 colorectal cancer patients who underwent R0 resection followed by postoperative 5-FU based adjuvant chemotherapy. Immunohistochemical examinations of NF-κB and JNK on tissue microarrays (TMAs) revealed that significantly shorter time-to-relapse (TTR) in both NF-κB(+) and JNK(−) subgroups in both gastric (NF-κB(+), *p* = 0.0002, HR11.7. 95%CI3 3.2–43.4; JNK(−), *p* = 0.0302, HR4.4, 95%CI 1.2–16.6) and colon (NF-κB(+), *p* = 0.0038, HR36.9, 95%CI 3.2–426.0; JNK(−), *p* = 0.0098, HR3.2, 95%CI 1.3–7.7) cancers. These protein expression patterns also show strong discriminately power in gastric cancer patients for overall survival rate, suggesting a potential utility as prognostic or chemosensitivity markers. Baseline expression of these proteins using gastric cancer cell lines demonstrated the reciprocal patterns between NF-κB and JNK, while 5-FU exposure of these cell lines only induced NF-κB, suggesting that NF-κB plays a dominant role in the response to 5-FU. Subsequent siRNA experiments confirmed that gene knockdown of NF-κB increased 5-FU-specific sensitivity, whereas that of JNK did not affect the chemosensitivity. These results suggest that the expression of these proteins may aid in the decisions involved with adjuvant chemotherapy for gastrointestinal tract cancers.

## Introduction

Although several standard chemotherapeutic regimens have been established, there is still a great need to identify chemosensitivity or prognostic markers that allow for the prediction of cancer chemotherapy efficacy. The application of biomarkers with high discriminatory power can help clinicians avoid difficult chemotherapy regimens with unnecessary adverse effects as well as allow for an earlier decision to use alternative regimens. However, despite the use of several high throughput screening methods in this context, the identification of biomarkers has been difficult [1].

During the characterization of molecular and cellular characteristics of a panel of 12 human cancer cell lines, we developed a system in which a conventional *in vitro* chemosensitivity assay using clinically approved drugs combined with quantitative protein expression profiling using a ‘reverse-phase’ lysate array (RPA) was used to identify proteins that may be relevant to the activity of the chemotherapeutic agents [2]. Both technologies produce a quantitative output, which allows for the analysis of a large number of combinations between drug potency and protein expression [2], [3]. Moreover, this system hypothesizes that the expression profile of a protein may be a predictor of chemosensitivity to a given drug. Subsequent validation is then required to determine if the markers are clinically relevant with regard to chemosensitivity.

A recent collection of individual patient data from colon cancer cases has revealed that 5-FU-based adjuvant chemotherapy provides a significant disease-free survival (DFS) benefit by reducing the recurrence rate, which leads to a long-term overall survival (OS) benefit [4]. In East Asian countries, it has been well-accepted that resectable, locally advanced gastric cancer will benefit from 5-FU-based adjuvant chemotherapy such as S-1, which is an oral fluoropyrimidine, for prolonged OS and recurrence-free survival (RFS) [5], [6]. However, approximately 30–40% of patients experience recurrence even after receiving a curative operation and ‘standard’ adjuvant chemotherapy [7], [8]. Despite the intensive use of 5-FU for gastrointestinal cancers, to-date the markers for 5-FU have not achieved standard-of-practice usefulness [9].

In the present study, we collected 79 surgically removed cancer specimens from gastric and colon cancer patients who had not received any chemotherapy at the time of operation and later received 5-FU based adjuvant chemotherapy to determine if any of the markers were associated with TTR. We produced a tissue microarray (TMA) representing all 79 specimens on a glass slide and probed them with primary antibodies [2] that recognized a specific protein identified as a candidate marker for relapse. To confirm a direct association of protein expression and the 5-FU anti-tumor effect, we also performed gene knockdown by siRNA in several human cancer cell lines.

## Materials and Methods

### Ethics Statement

The study has been approved by Institutional Review Board at Iwate Medical University in compliance with the Helsinki declaration. An individual written consent was obtained from all patients and the absolute confidentiality was preserved even after the patient has died. All analyses were performed anonymously so individual patients were not identified.

### Prediction of Proteins as Candidate Markers of Prognosis

**Figure 1:**
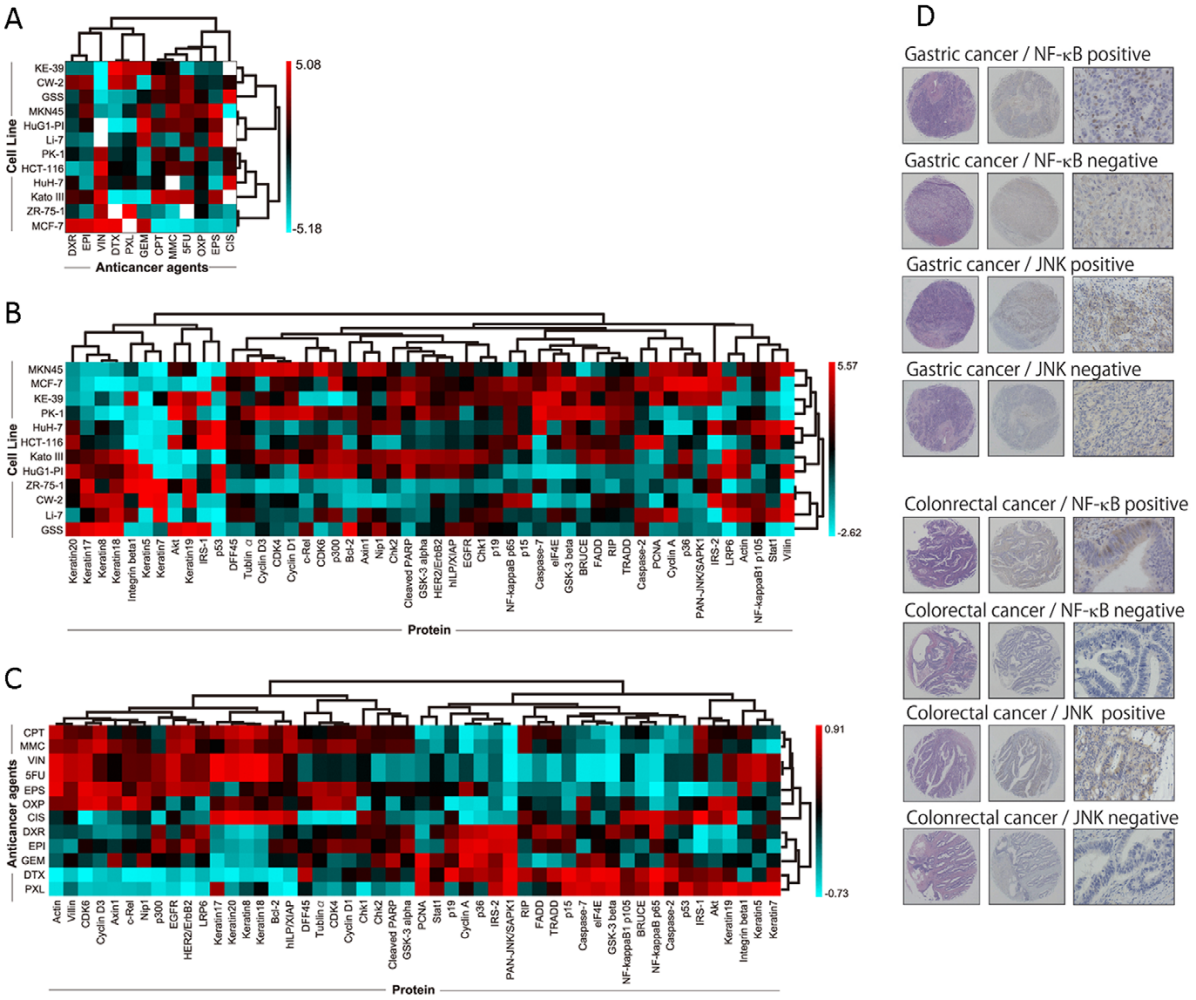
Hierarchical clustering of three different matrices and results of immunohistochemical examinations of candidate markers. (A) Based on a chemosensitivity assay of a cancer cell line panel, the A (activity) × C (cells)  =  AC matrix was created. (B) Quantitative protein expression data of each cell line determined by “reverse-phase” lysate microarray generates the C×P (protein)  =  CP matrix. (C) A heatmap with hierarchical clustering representation of the AP matrix, which is generated from AC and CP matrices. (D) Immunohistochemical stainings of candidate markers for 5-FU treatment.

We first performed a conventional chemosensitivity assay whereby 144 combinations of 12 anticancer drugs and 12 cell lines were evaluated for chemosensitivity using a 50% growth inhibition (GI_50_) value (A matrix, Fig. 1A) [2]. The baseline expression level of 50 proteins from the cell line panel was quantitatively analyzed using a ‘reverse-phase’ lysate microarray (RPA) [10], [11], which is a western blot in microscale dot format, followed by quantitative immunodetection (P matrix) [12], where each matrix is visualized based on average-linkage hierarchical clustering (Fig. 1B) [2], [13]. A correlation of correlation between A and P matrices (AP matrix) was then established using the algorithm reported by Scherf et al. [14] (Fig. S1, S2). The AP matrix allows us to predict an association between protein expression and chemosensitivity. Using this method, we identified eight proteins based on 5-FU chemosensitivity (Fig. 1C, Table S1) [2].

### Surgically Removed Specimens

Seventy-nine surgically removed specimens, including 30 gastric and 49 colorectal cases, were collected from patients who had not received any anticancer agents by the time of surgery. All surgical cases were conducted at the Department of Surgery at Iwate Medical University Hospital between 1997 and 2008. After the surgery, all cases were confirmed to meet the criteria for 5-FU-based adjuvant chemotherapy together with a final clinicopathological diagnosis (Table 1).

**Table 1:** Postoperative Clinicopathological Characteristics in Cancer Patients for 5-FU Based Chemotherapy.

Characteristic	Total (n = 79)		Stomach (n = 30)		Colorectal (n = 49)	
	No.	%	No.	%	No.	%
Age, years						
Median	68		68		68	
Range	37–83		45–83		37–80	
Sex						
Male	49	62	19	63	30	61
Female	30	38	11	37	19	39
T factor						
High	53	67	16	53	37	76
Low	26	33	14	47	12	24
N factor						
High	53	67	28	93	25	51
Low	26	33	2	7	24	49
Stage						
High	42	53	17	57	25	51
Low	37	47	13	43	24	49
†Chemotherapy Completed						
Completed	60	76	25	83	35	72
Suspended	15	19	5	17	10	20
Unknown	4	5	0	0	4	8

TN factors and Stages are divided into the following binary categories: High (≥T3), and Low (T2); High (≥N1), and Low (N0); and High (≥Stage III), and Low (Stage I, II).

† Chemotherapy completed, continued chemotherapy for 0.5 years for colorectal and 1 year for stomach. Information on chemotherapy completion was not available in four cases. NA, not applicable.

### TMAs

A tumor-rich area of each tissue specimen was marked on a hematoxylin and eosin (H&E) stained section under a microscope. The core cylinder of the tumor-rich area from each specimen was punched out from the paraffin block using a manual tissue microprocessor (KIN-1, Azumaya, Japan) with a steel needle having an inner diameter of 2 mm [15]. The cylindrical cores were arrayed in recipient paraffin blocks. TMA sections (4 µm thick) were obtained using a standard preparation. In the present study, a core was punched out for each sample in the paraffin block, but some adjacent sections of strongly positive or negative samples were also examined to assess for any considerable heterogeneity.

### Immunohistochemistry

The tissue specimens were incubated at 97°C in 1 mM EDTA pH 9.0 for 30 min in a microwave oven for antigen retrieval. They were then incubated with the primary antibodies NF-κB p65 (Cell Signaling Technology, Danvers, MA) and JNK/SAPK1 (BD BioSciences, Franklin Lakes, NJ) overnight at 4°C. Immunostaining was performed using a DAKO Envision+ system (DakoCytomation, Denmark) and an autostainer. For NF-κB staining evaluation, samples with more than 10% clear nuclear staining were designated as positive (Fig. 1D). For JNK staining evaluation, we first divided the staining strength into 4 grades, and subsequently divided them into two groups (negative and positive). The staining evaluation was focused on the nucleus for NF-κB, and on the cytoplasm for JNK, in epithelial components for both stainings [16]. Information on the primary antibodies used in the study is provided in Table S2. The final staining score was tabulated in a binary manner for statistical analyses.

### Statistical Analysis

Associations between clinicopathological characteristics and immunohistochemical data were used to evaluate the significance among categorical variables by the Fisher’s exact test or χ^2^ test. TTR and OS were calculated from the date of operation to either the date of relapse, death, or censoring with Kaplan-Meier estimation by grouping protein expression scores. A Kaplen-Meier estimator between groups of immunohistochemical grades was compared with a log-rank test. Multivariate subset analysis using a Cox proportional hazard model was performed to explore the interaction between TTR/OS and to identify independent factors. All statistical analyses were performed using JMP 7.0 (SAS Institute, Cary, NC).

### Molecular Marker Induction by 5-FU

Five human gastric cancer cell lines (GSS, HuG1-PI, KATOIII, KE39, and MKN45) were grown in RPMI1640 supplemented with 10% FBS. Baseline protein expression levels of the identified markers (NF-κB/p65 and JNK) were measured by western blot. Cells were trypsinized for cell lysate preparation according to a published protocol [17]. Nitrocellulose membranes were then incubated with the primary antibodies used for immunohistochemistry followed by chemoluminescent signal development (SuperSignal West Pico, Thermo Scientific, USA). To determine if the respective protein levels were enhanced by 5-FU, two different concentrations of 5-FU (50 and 100 µM) were added in the culture medium for four hours. Immunocytochemistry was also performed on four-chamber cell culture slides using the same primary antibodies described above followed by either Alexa488 or 564-conjugated secondary antibodies, respectively. A standard fluorescent microscope was used to examine the cellular localization of the proteins.

### Target Gene Knockdown

Five human gastric cancer cell lines were grown to 70% confluency in RPMI1640 supplemented with 10% FBS in a 96-well plate. The cells were then treated with a cationic-lipofection reagent (*Trans*IT-TKO Transfection Reagent, Mirus, WI) in the presence of siRNA specific for either NF-κB p65 or JNK gene transcripts (Signal Silence, Cell Signaling Technologies, MA) for 48 h. After the siRNA transfection, each drug (5-FU, cisplatinum, docetaxel, and paclitaxel) was added at a concentration that inhibited 50% cell growth (GI_50_) for each cell line [2] and then incubated for an additional 48 h for the growth inhibitory assay (WST-1, Dojindo, Japan). The effect of NF-κB on growth suppression was evaluated if the growth was reduced to less than 50% by siRNA with a drug concentration at the GI_50_. The effect of JNK siRNA was evaluated if cell growth was more than 50% of the control. All experiments were repeated at least three times. To verify the gene specific effect of siRNA, we have also performed an experiment using different siRNAs targeting p65 and JNK (SignalSilence siRNAI and siRNAII for NF-κB and SAPK/JNK, respectively; Cell Signaling Technology). The same trend was obtained for both siRNAs. Control samples were corresponding cell lines with siRNA transfection without anticancer drugs.

## Results

### Patients

The median age of the 79 patients was 68 years (range, 37–83 years), with 30 gastric cancer and 49 colorectal cancer patients. All 79 patients underwent either a gastrectomy or colorectomy with lymph node dissection. The operational curability was no residual tumor (R0) for all cases. Pathological findings revealed that all tumors invaded beyond the mascularis propria. Twenty-six (33%) of the patients had pathologically negative regional lymph node metastases, while no patients showed distant metastases. All patients satisfied the following criteria for 5-FU-based adjuvant chemotherapy: Histologically confirmed gastric (> Stage II) or colorectal (>T2) cancer with apparent R0 surgery; no hepatic, peritoneal, or distant metastasis; patient age between 20 and 85; no prior chemotherapy; and adequate organ function. Chemotherapy was considered to be completed if a patient was able to continue the following 5-FU based regimens: (i) S-1 (60 mg/m^2^/body) for 1 year for gastric cancer; and (ii) either doxifluridine (800–1200 mg/day), 5-FU (370 mg/m^2^/day), or UFT-E granules (300 mg/m^2^/day) for six months for colorectal cancer. Sixty (76%) patients completed chemotherapy, 15 (19%) patients suspended treatment, and 4 (5%) patients had missing information.

The median observation time after operation was 3.41 and 5.04 years in stomach and colon, respectively (range, 1.41–7.00 years in stomach; and 0.99–10.24 years in colon). In non-relapsed cases, the minimum observation time was 2.43 and 2.36 years in stomach and colon, respectively. The median TTR was 1.56 and 1.52 years in stomach and colon, respectively (range, 0.72–3.33 years in stomach; and 0.27–4.47 in colon). Among 77 cases of which survival status was confirmed, the 3-year overall survival rate in the relapsed and non-relapsed groups was 0.60 and 0.65 in stomach and 0.97 and 0.96 in colon, respectively. The clinicopathological parameters on the basis of relapse status are shown in Table S3.

### Immunohistochemistry

The immunostaining scores of all candidate proteins were evaluable (Fig. 1D and Fig. S3). NF-κB showed distinct nuclear staining that was scattered throughout the nuclei and did not form clusters. Some cells showed cytoplasmic staining, but it was not as distinct as those with nuclear staining. The staining of JNK was not as strong as that of NF-κB but was clearly localized in the cytoplasm. The remaining six candidate proteins and three proteins of interest were in their expected subcellular locations (Fig. S3). After determining the subcellular localization of the proteins, the strength of staining was scored in a binary manner.

### Correlation between Protein Expression and Clinicopathological Findings

A contingency parameter analysis of each protein in terms of relapse revealed that the protein levels of NF-κB and JNK were significantly associated with relapse in stomach (*p* = 0.0004 and 0.029 for NF-κB and JNK, respectively; Table S4). When the expression of these two proteins was combined, the contingency analysis demonstrated a stronger discriminating power than each individual protein.

**Figure 2:**
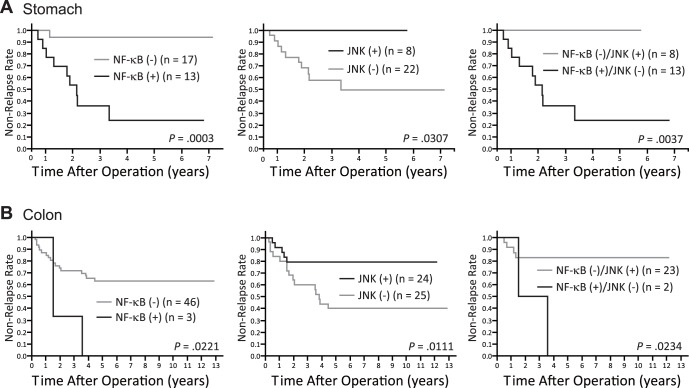
Time-to-relapse (TTR) rates on the basis of NF-κB and JNK protein expression in gastric and colon carcinomas.

Based on a Kaplan-Meier analysis, JNK and NF-κB expression levels were associated with significant differences in the non-relapse rate (Fig. 2). A log-rank test of the Kaplan-Meier analysis showed a significant difference in the non-relapse rates between the JNK(+) and JNK(−) groups in both stomach (*p* = 0.0302, HR4.4, 95%CI 1.2–16.6) and colon (*p* = 0.0098, HR3.2, 95%CI 1.3–7.7); and also between the NF-κB(+) and NF-κB(−) groups in both stomach (*p* = 0.0002, HR11.7, 95%CI 3.2–43.4) and colon (*p* = 0.0038, HR36.9, 95%CI 3.2–426.0, Fig. 2). Interestingly, in stomach, all NF-κB(+) cases were JNK(−), whereas 58% of JNK(−) cases were NF-κB(+). The probability of relapse when these markers were combined showed greater difference than using the individual markers in both stomach and colon (Fig. 2).

We also screened p53, Tymidine Synthetase, and MDR-1 expression in pooled stomach and colon samples because it has been suggested that these proteins or encoding genes may be associated with 5-FU drug potency [18], [19], [20], [21]. However, no significant association was observed between the relapse rate and the expression level of these proteins (Fig. S4).

Of the 3-year OS rate of gastric cancer, NF-κB(−) and (+) cases was 0.94 and 0.77, respectively, and 0.82 and 1.00 for JNK(−) and (+), respectively. Of the 3-year OS rate of colon cancer, NF-κB(−) and (+) cases was 0.79 and 1.00, respectively, and 0.72 and 0.90 for JNK(−) and (+), respectively. However, there was a significant difference in the OS rate by Kaplan-Meier estimation in gastric cancer (NF-κB(+), HR 7.9, 95%CI 2.1–30.3; JNK(−), HR 0.25, 95%CI 0.06–1.09, Fig. S5).

### Subset Analysis

To identify general relationships between markers and clinicopathological findings, a subset analysis was performed with stomach and colorectal pooled samples. There was a significant association between NF-κB status and T-factor/Stage for TTR (Fig. S6), but no significant association was observed between JNK status and any variables for TTR (Fig. S7). Interestingly, however, there was a significant association between the combined marker status: and T-factor/Stage for TTR, and chemotherapy completion status for OS (Fig. S8, S9). The association between OS and the status of each factor was also analyzed according to sex, age, lesions, TNM classifications, and the status of chemotherapy completion (Fig. S10, S11). A multivariate analysis revealed that NF-κB expression and the T factor were independent factors for both relapse and survival.

### Molecular Responses by 5-FU

**Figure 3:**
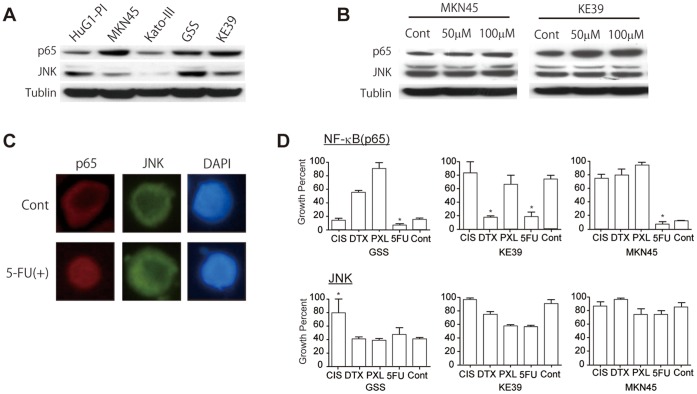
Induction of biomarkers by 5-FU treatment. (A) Baseline protein expression of NF-κB and JNK in five gastric cancer cell lines. Tublin was used as a loading control. (B) Induction of candidate biomarkers in response to 5-FU treatment in different concentrations in MKN45 and KE39. (C) Examination of protein localization by fluorescent immunocytochemistry using MKN45. (D) Increased inhibitory growth effect by anticancer agents in gastric cancer cell lines after transfection of siRNA for NF-κB p65 and JNK transcripts. Control samples are the corresponding cell lines transfected with the indicated siRNAs without anticancer agents. Abbreviations are: CIS, cisplatinum; DTX, docetaxel; and PXL, paclitaxel; and 5FU, 5-fluorouracil. *p<0.05, Student *t*-test.

Baseline protein expression of NF-κB and JNK was measured by western blot. Interestingly, the expression pattern was reciprocal; cell lines with high NF-κB expression showed relatively low JNK expression (Fig. 3A). The reciprocal expression pattern was concordant with the directionality of these proteins as biomarkers. We also tested the protein induction by 5-FU. NF-κB expression was induced and increased in the total protein fraction by 5-FU in a dose-dependent manner (Fig. 3B). Subsequent immunocytochemical analysis revealed that nuclear NF-κB was prominently visualized after 5-FU exposure, while JNK did not exhibit a noticeable change in localization (Fig. 3C).

### The Effect of NF-κB p65 and JNK Gene Knockdown on Cell Growth

Four out of five cell lines (GSS, KATOIII, KE39, and MKN45) exhibited significant growth suppression after NF-κB siRNA transfection and 5-FU treatment (*p*<0.05, Student *t*-test; Fig. 3D, Fig. S12). The 5-FU-dependent, statistically significant growth suppression was seen in GSS, KATO-III, and MKN45. JNK siRNA treatment was expected to induce an anti-apoptotic effect based on previous studies [22], which was hypothesized to increase the growth rate in the presence of anticancer drugs. Four out of five cell lines treated with JNK siRNA demonstrated 5-FU-mediated growth induction or no change. These siRNA experiments revealed that NF-κB and JNK seem to have reciprocal roles in terms of 5-FU-mediated growth suppression.

## Discussion

Although the choice of adjuvant chemotherapy after resection of gastric cancer is slightly different between countries, 5-FU has been shown to be the most effective treatment option. Validation of postoperative chemotherapy and surgery alone has demonstrated 3-year OS rates of 80.1% and 70.1%, respectively, in Japan [23]. Postoperative chemoradiotherapy (CRT) has been conducted in the United States for advanced gastric cancer, and the OS after CRT has been reported to be 50%, while that of surgery alone was 41% [24]. In Europe, the MAGIC trial revealed the efficacy of perioperative chemotherapy and demonstrated that the 5-year OS was 36.3% and 23.0% in the perioperative chemotherapy and surgery alone groups, respectively [25]. On the other hand, adjuvant chemotherapy (5-FU/LV) for advanced colorectal cancer was shown to improve OS in the 1990s, and more recently a further improvement of DFS and OS has been observed with the addition of oxaliplatin [26]. In general, adjuvant chemotherapy for colorectal cancer is only administrated for six months, but has been shown to provide long-term benefits, including prolonged 5-year DFS and OS [4], [26].

With a standard of care established, we now face the issue of how to treat patients who fail these standard therapies. In fact, 30–40% of patients treated with the standard adjuvant chemotherapy experience recurrence after surgery within five years in advanced gastric and colon carcinomas [8], [27]. Therefore, it has been an important goal to improve treatment regimens for the subset population where standard therapy may be ineffective.

In the present study, to validate the utility of identified markers, we collected archived tissues from patients that had undergone curative resection for gastric and colorectal cancer. All patients were eligible to receive adjuvant chemotherapy, including patients with stage I/II colorectal cancer. A previous report showed that among 769 mp cases from a retrospective cohort of Japanese patients, the recurrence rate of stage I (mp, N0) patients was 7% [28]. Although the number of patients was limited, the QUASAR study reported that a higher percentage of stage I patients (25%) who had received surgery alone died within five years compared to patients who received adjuvant chemotherapy [29]. Moreover, it has been reported that circulating tumor cells were found in 6% of stage I colorectal cancer patients after curative operation [30]. These clinical and biological findings should make it reasonable the inclusion of Stage I in the present study.

It has been reported that approximately 25% of patients with stage II tumors are considered to have an increased risk of recurrence because of: (a) penetration of the serosa (T4); (b) extramural venous invasion; (c) poorly differentiated histology; (d) presentation of an obstruction; or (e) having a yield of less than 10–12 lymph nodes [31]. In the present study, most of the stage II colorectal cases possessed one of these risk criteria, except in one case where the patient was young (37-years-old), for which the QUASAR study justified the eligibility. Although the use of chemotherapy in stage I is not recommended and Stage II cases has been controversial, we conducted the validation because it aimed to select an individual who may not benefit from “standard” chemotherapy or have a potential risk in lower stages, which is in contrast to the approach of epidemiological studies.

Previous reports have demonstrated the significance of NF-κB in prognosis, angiogenesis, and chemoresistance in stomach and colon carcinomas [32], [33], [34], [35], [36]. Our present data demonstrated that the nuclear localization of NF-κB could predict the outcome of patients at the time of operation who subsequently receive adjuvant chemotherapy. Interestingly, NF-κB(+) tumor cells were found scattered in the sections in which there was a clear distinction between positive and negative cells. Molecular experiments revealed a clear reciprocal relationship between NF-κB and JNK expression, which suggested a potential association with 5-FU therapy and the pathological findings. To clarify the role of NF-κB and JNK in the tumor response to 5-FU, we conducted gene knockdown experiments. Knockdown of the NF-κB (p65) gene revealed that the majority of cancer cell lines tested demonstrated clear 5-FU-specific growth suppression, while other drugs even induced cell growth. This result suggests a direct association between 5-FU sensitivity and NF-κB expression and supports the diagnostic application of this analysis for 5-FU-based adjuvant chemotherapy. It should also be noted that taxans and topoisomerase inhibitors activate the NF-κB pathway, which leads to cell proliferation through MYC and IKK activation, respectively [33]. The clinical implications of these mechanisms remain to be elucidated.

NF-κB has been implicated in the development of drug resistance in a wide range of cancer cells. Inhibition of NF-κB activation reduced chemoresistance in gastric and colorectal cancer cell lines, which is consistent with our present results [6], [32], [37], [38]. Constitutive activation of NF-κB has been suggested as a potential prognostic factor in gastric cancer [39], [40], [41] and correlates with the progression and chemotherapy resistance of colorectal carcinomas [42], [43].

JNK proteins have diverse functions on cell proliferation and on the induction of apoptosis through stress-activated protein kinase pathways, and are often down-regulated in cancers [44], [45]. In the present study, the role of JNK in the context of 5-FU response seems to be passive with respect to chemosensitivity, according to our siRNA experiment. The immunohistochemical analysis in this study showed that NF-κB and JNK were reciprocal indicators of prognosis. However, knockdown of NF-κB sensitized 5-FU, while JNK did not make cells resistant to 5-FU. NF-κB activation by TNF-α is tightly regulated by JNK in the context of a proinflammatory response, which occurs immediately after stimulation [46], [47], [48], [49]. On the other hand, activation of NF-κB in malignancy or chemoresistance seems to be constitutive in a part of gastrointestinal tumor progression [47], [50]. In the present study, although NF-κB nuclear staining was only seen in a small fraction of tumor cells, JNK was stained relatively ubiquitously throughout the tissue. Therefore, our current results may indicate that JNK staining reflects a degree of background chronic inflammatory or stress responses of gastric mucosa [51], while NF-κB constitutive activation is associated with the malignant potential of the tumor cells [39], [40], [41], [52]. In addition to the intrinsic malignant potential, our results also demonstrated that NF-κB plays a specific role in 5-FU response. Taken together, although a larger clinical research is required, NF-κB nuclear expression may be a good candidate as a 5-FU chemosensitivity prediction marker, while JNK may be a supportive marker that reflects the background mucosal information.

Although the present result is still preliminary from a practical point of view, these results may provide an opportunity for alternative regimens to be considered for cases that indicate a low probability of a 5-FU-based chemotherapeutic response. A larger immunohistochemical study that includes NF-κB/JNK analyses will be necessary to prove the utility in gastric and colorectal cancers.

## Supporting Information

Figure S1 **Flow of Chemosensitivity Marker Identification.** (A) Based on a chemosensitivity assay of a cancer cell line panel, the A (activity) × C (cells)  =  AC matrix was created. The left two panels show cell growth curves on the basis of drug concentration. The middle panel shows the 50% growth inhibition (GI_50_) values in a bar graph. All data are centered by Peak Plasma Concentration (PPC) values that are unique for each drug. The right panel represents the GI_50_ values and the cells in a heatmap with a hierarchical clustering format. (B) “Reverse-phase” protein lysate microarray (left) and C (cells) × P (protein)  =  CP matrix in a heatmap with a hierarchical clustering format (right). (C) A heatmap with hierarchical clustering representation of the AP matrix, which is generated from AC and CP matrices. The dendrogram indicates the distance based on the correlation coefficient of the data set next to each other. Hence, the AP matrix shows the correlation between protein expression and drug efficacy across all cell lines. Cited with permission from reference #2.
(TIF)

Figure S2 **Correlation between candidate proteins and drug sensitivity.** Left: Scattergram based on 5-FU sensitivity and NF-κB expression. The correlation coefficient is positive, but is negative (*r* = −0.304) when the gastrointestinal cell lines (CW2, HCT116, GSS, KATOIII, MKN45, HuG1-PI, and KE39) were analyzed, which is consistent with the validation result from the TMAs. Right: Scattergram based on 5-FU sensitivity and JNK expression. It has been well-accepted that screening tools, such as microarray-based techniques, can discover useful biomarkers, but may also isolate false-positives. The correlation coefficient of NF-κB and drug sensitivity was positive for the screening, which was expected to identify a trend whereby higher protein expression correlated with higher drug sensitivity; however, the result was opposite. A possible explanation for the discrepancy is that the number of cell lines for the screening may be too small. In fact, most of the gastrointestinal cell lines lined up as a “negative slope”, which is consistent with the clinical result. As expected, subsequent confirmation molecular analysis revealed the association between NF-κB and 5-FU.
(TIF)

Figure S3 **Immunohistochemical staining of candidate proteins on TMAs.** The TMAs were used to validate expression of 9 proteins. Each protein shows a set of 6 panels. The top rows represent positive staining, while the bottom row represents the corresponding negative samples. From the left, H&E staining (40x), a low power immunohistochemical image (40x), and a high power immunohistochemical image (400x). The level of staining for each specimen was scored in a binary manner.
(TIF)

Figure S4 **Time-to-relapse (TTR) on the basis of candidate protein expression.** TTP was compared on the basis of candidate protein expression in a binary manner from immunohistochemical staining of the TMAs. There were 79 patients assessed, including both gastric and colorectal cancer patients.
(TIF)

Figure S5 **Kaplan-Meier estimation of the non-relapse rate and Overall Survival (OS), depending on the lesions, based on NF-κB and JNK expression.** (A) Stomach, and (B) Colon.
(EPS)

Figure S6 **Hazard ratio for relapse and p values for the interaction of NF-κB status and clinical subgroup categories.**
(TIF)

Figure S7 **Hazard ratio for relapse and p values for the interaction of JNK status and clinical subgroup categories.**
(TIF)

Figure S8 **Hazard ratio for relapse and p values for the interaction of NF-κB/JNK status and clinical subgroup categories.**
(TIF)

Figure S9 **Hazard ratio for death and p values for the interaction of NF-κB/JNK status and clinical subgroup categories.**
(TIF)

Figure S10 **Hazard ratio for death and p values for the interaction of NF-κB status and clinical subgroup categories.**
(TIF)

Figure S11 **Hazard ratio for death and p values for the interaction of JNK status and clinical subgroup categories.**
(TIF)

Figure S12 **Enhanced growth inhibitory effect by p65 gene knock down.** Growth inhibitory effect of anticancer drugs at a concentration that elicits a 50% growth inhibitory (GI50) effect after 48 h of incubation in gastric cancer cell lines after transfection of siRNA for NF-κB p65 subunit (A) and JNK (B).
(TIF)

Table S1 **Candidate Markers Identified From Quantitative Protein Expression Analysis and Chemosensitivity Assay**
(DOC)

Table S2 **Primary antibodies Used for Candidate Marker Validation on TMAs**
(DOC)

Table S3 **Clinicopathological Features of the State of Relapse**
(DOC)

Table S4 **Clinicopathological Features of Immunohistochemical Status**
(DOC)
