# Supplementary figures and images for: Molecular Marker Identification for Relapse Prediction in 5-FU-Based Adjuvant Chemotherapy in Gastric and Colorectal Cancers
Source: PLoS One. 2012 Aug 14;7(8):e43236. doi: 10.1371/journal.pone.0043236 (PMC3419205; doi:10.1371/journal.pone.0043236)

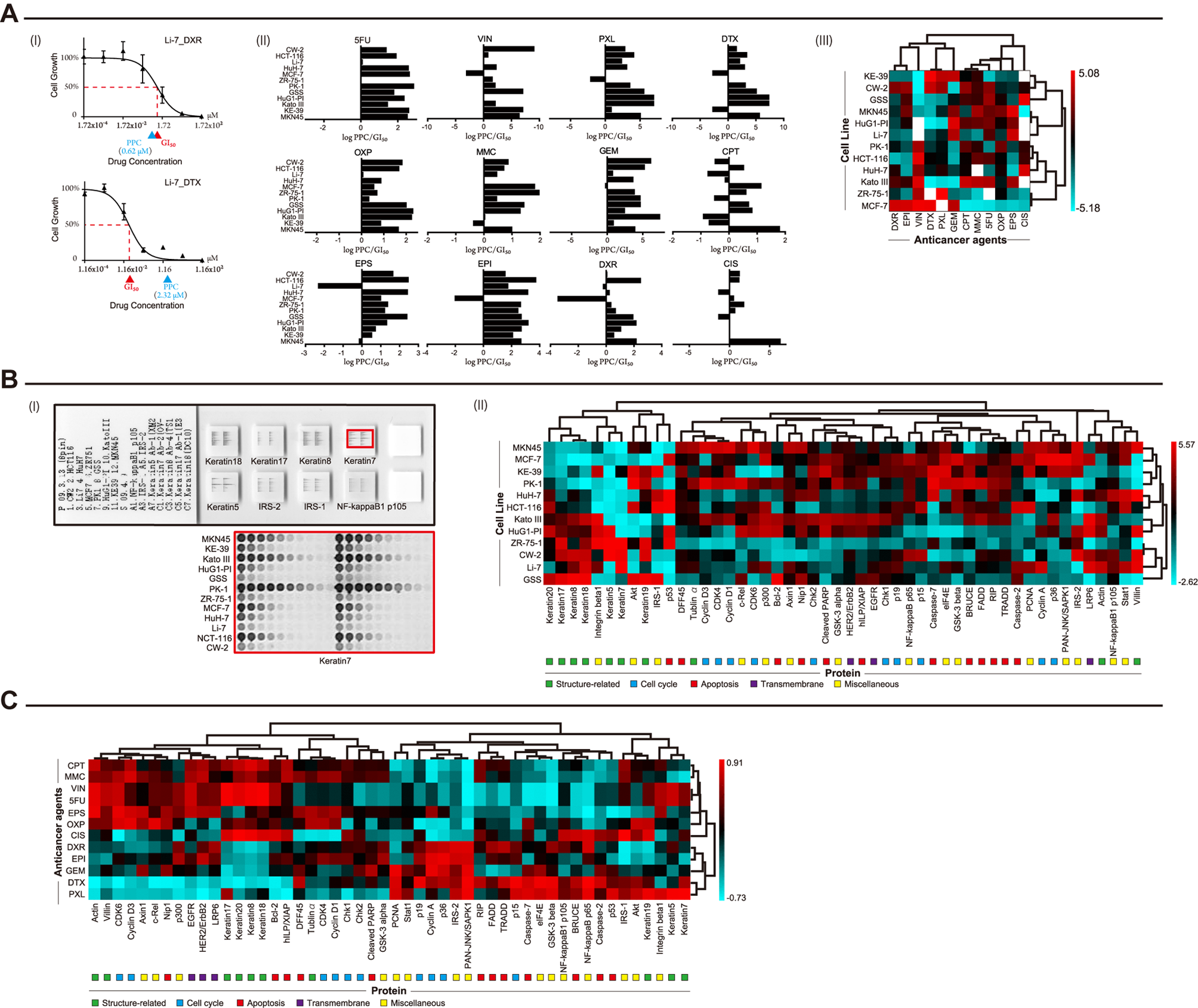

Supplement: Figure S1 — Flow of Chemosensitivity Marker Identification. (A) Based on a chemosensitivity assay of a cancer cell line panel, the A (activity) × C (cells) = AC matrix was created. The left two panels show cell growth curves on the basis of drug concentration. The middle panel shows the 50% growth inhibition (GI50) values in a bar graph. All data are centered by Peak Plasma Concentration (PPC) values that are unique for each drug. The right panel represents the GI50 values and the cells in a heatmap with a hierarchical clustering format. (B) “Reverse-phase” protein lysate microarray (left) and C (cells) × P (protein) = CP matrix in a heatmap with a hierarchical clustering format (right). (C) A heatmap with hierarchical clustering representation of the AP matrix, which is generated from AC and CP matrices. The dendrogram indicates the distance based on the correlation coefficient of the data set next to each other. Hence, the AP matrix shows the correlation between protein expression and drug efficacy across all cell lines. Cited with permission from reference #2. (TIF) [file pone.0043236.s001.tif]

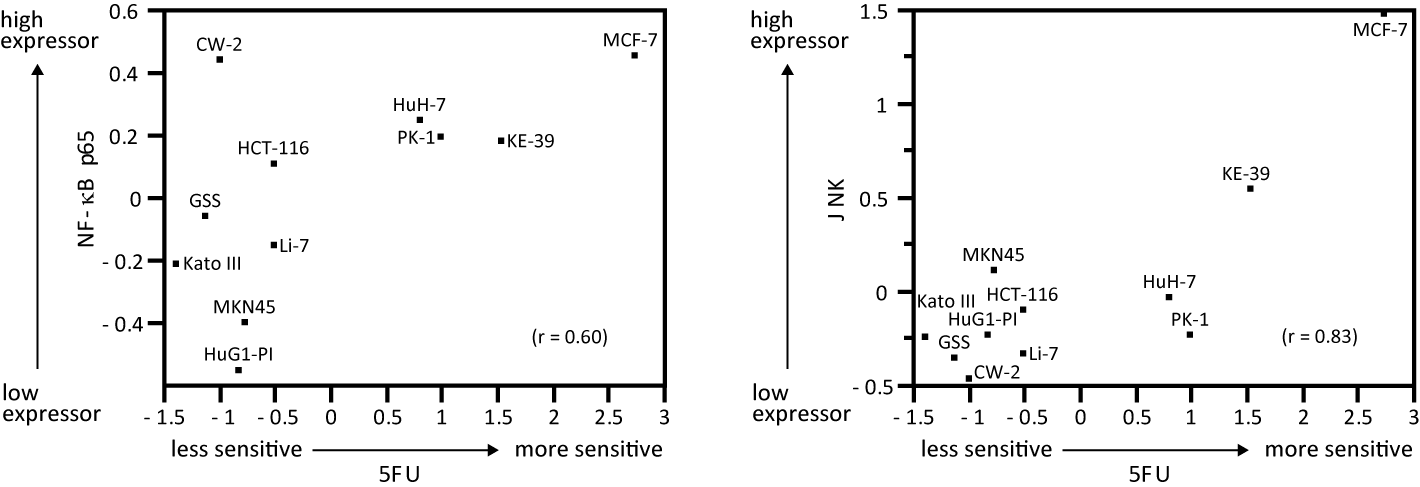

Supplement: Figure S2 — Correlation between candidate proteins and drug sensitivity. Left: Scattergram based on 5-FU sensitivity and NF-κB expression. The correlation coefficient is positive, but is negative (r = −0.304) when the gastrointestinal cell lines (CW2, HCT116, GSS, KATOIII, MKN45, HuG1-PI, and KE39) were analyzed, which is consistent with the validation result from the TMAs. Right: Scattergram based on 5-FU sensitivity and JNK expression. It has been well-accepted that screening tools, such as microarray-based techniques, can discover useful biomarkers, but may also isolate false-positives. The correlation coefficient of NF-κB and drug sensitivity was positive for the screening, which was expected to identify a trend whereby higher protein expression correlated with higher drug sensitivity; however, the result was opposite. A possible explanation for the discrepancy is that the number of cell lines for the screening may be too small. In fact, most of the gastrointestinal cell lines lined up as a “negative slope”, which is consistent with the clinical result. As expected, subsequent confirmation molecular analysis revealed the association between NF-κB and 5-FU. (TIF) [file pone.0043236.s002.tif]

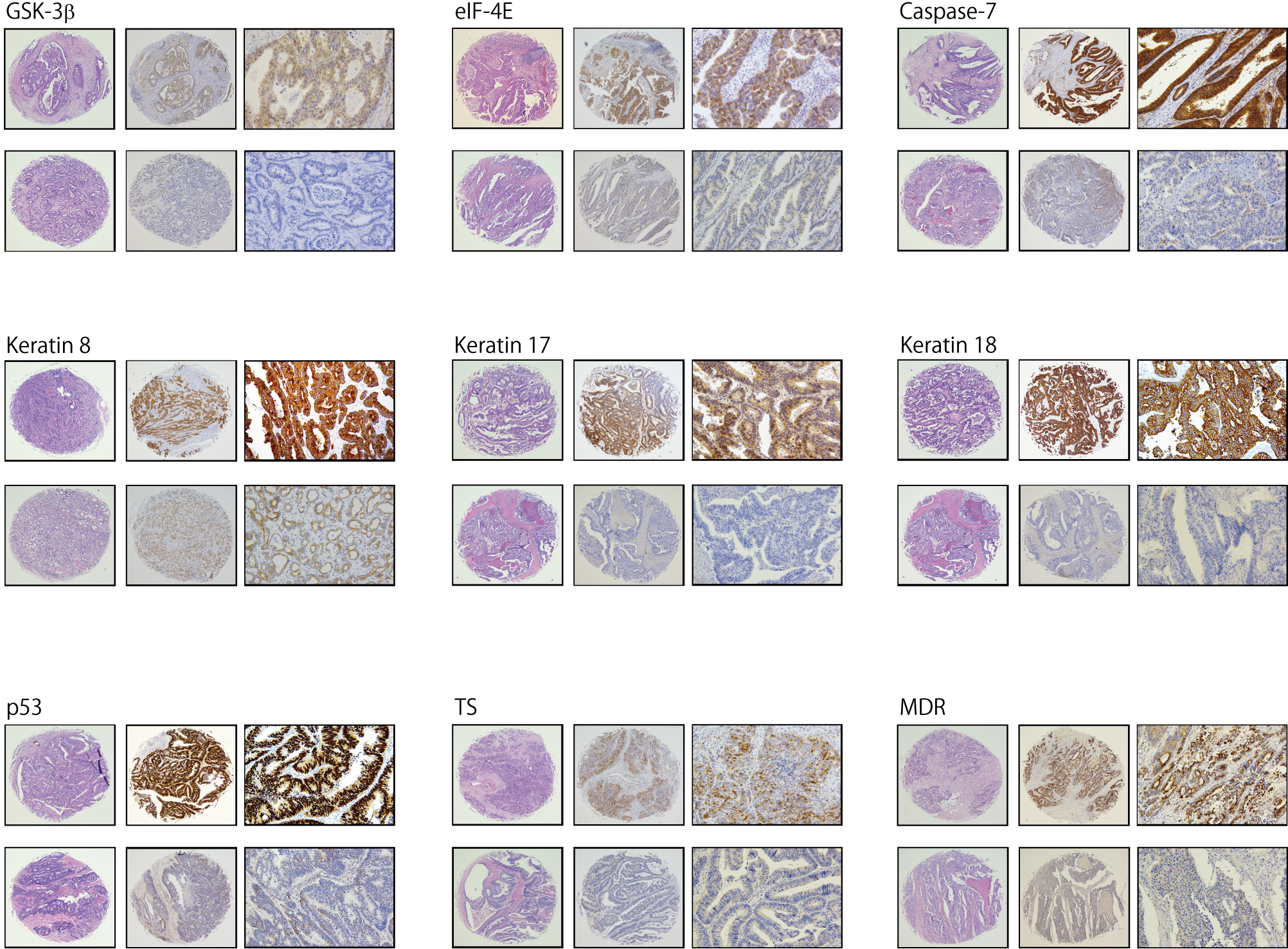

Supplement: Figure S3 — Immunohistochemical staining of candidate proteins on TMAs. The TMAs were used to validate expression of 9 proteins. Each protein shows a set of 6 panels. The top rows represent positive staining, while the bottom row represents the corresponding negative samples. From the left, H&E staining (40x), a low power immunohistochemical image (40x), and a high power immunohistochemical image (400x). The level of staining for each specimen was scored in a binary manner. (TIF) [file pone.0043236.s003.tif]

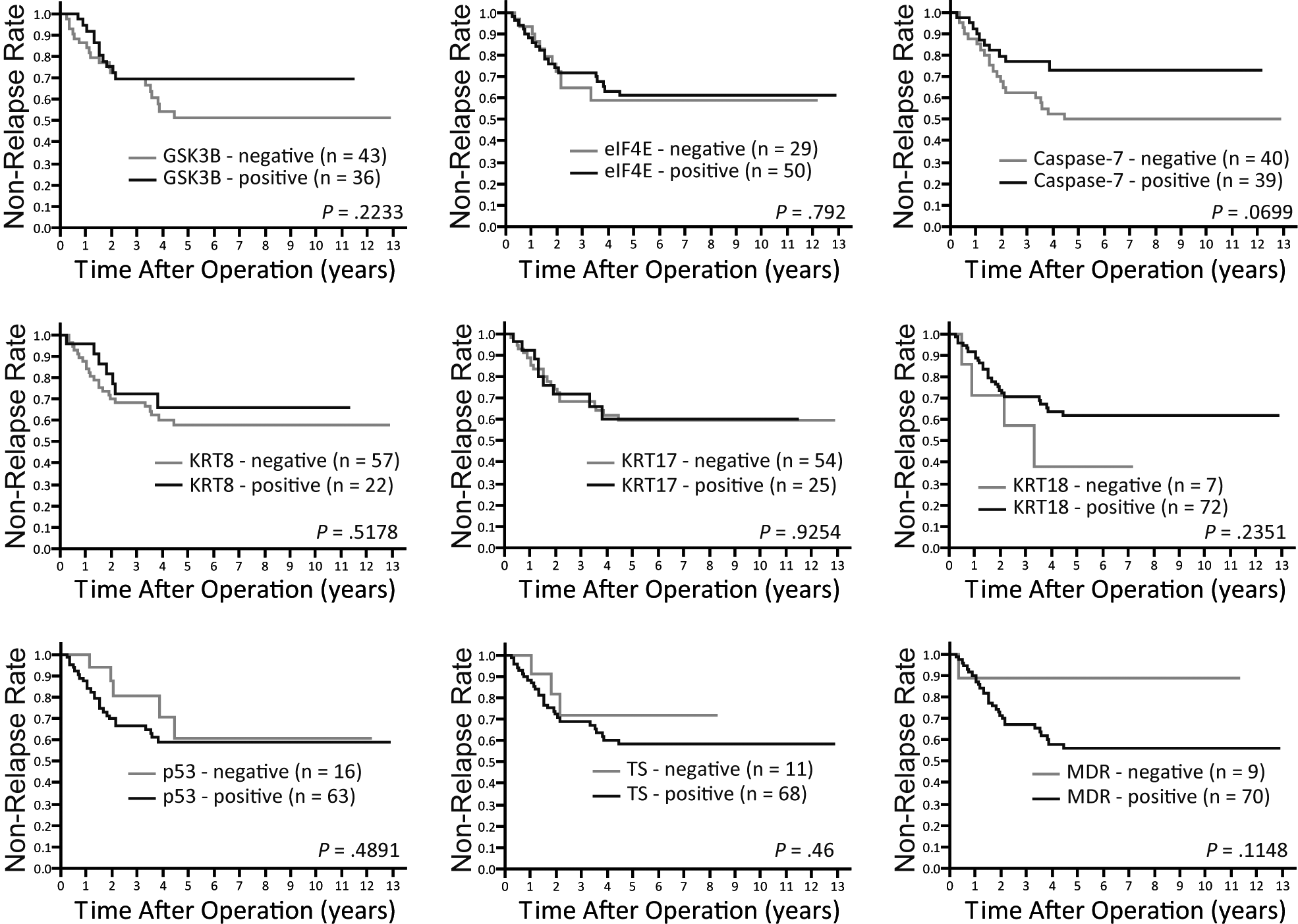

Supplement: Figure S4 — Time-to-relapse (TTR) on the basis of candidate protein expression. TTP was compared on the basis of candidate protein expression in a binary manner from immunohistochemical staining of the TMAs. There were 79 patients assessed, including both gastric and colorectal cancer patients. (TIF) [file pone.0043236.s004.tif]

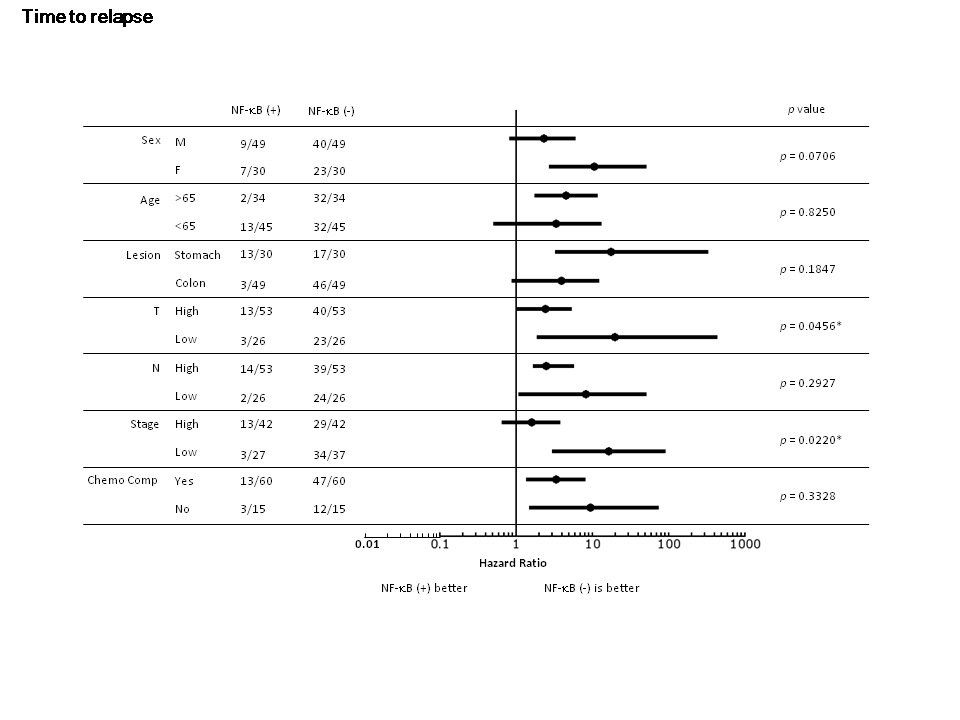

Supplement: Figure S6 — Hazard ratio for relapse and p values for the interaction of NF-κB status and clinical subgroup categories. (TIF) [file pone.0043236.s006.tif]

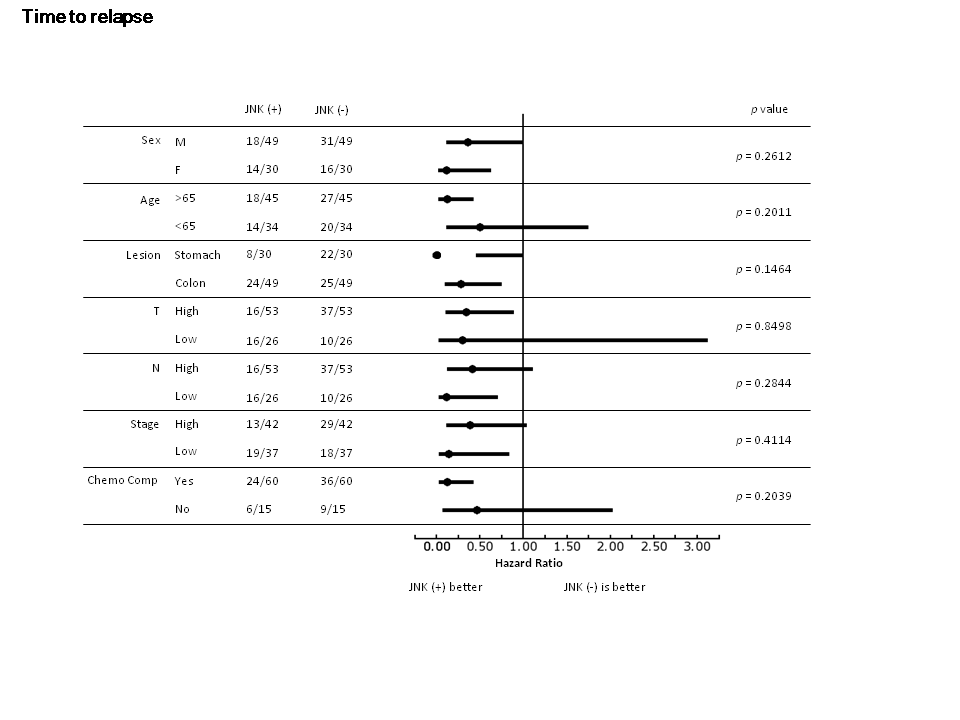

Supplement: Figure S7 — Hazard ratio for relapse and p values for the interaction of JNK status and clinical subgroup categories. (TIF) [file pone.0043236.s007.tif]

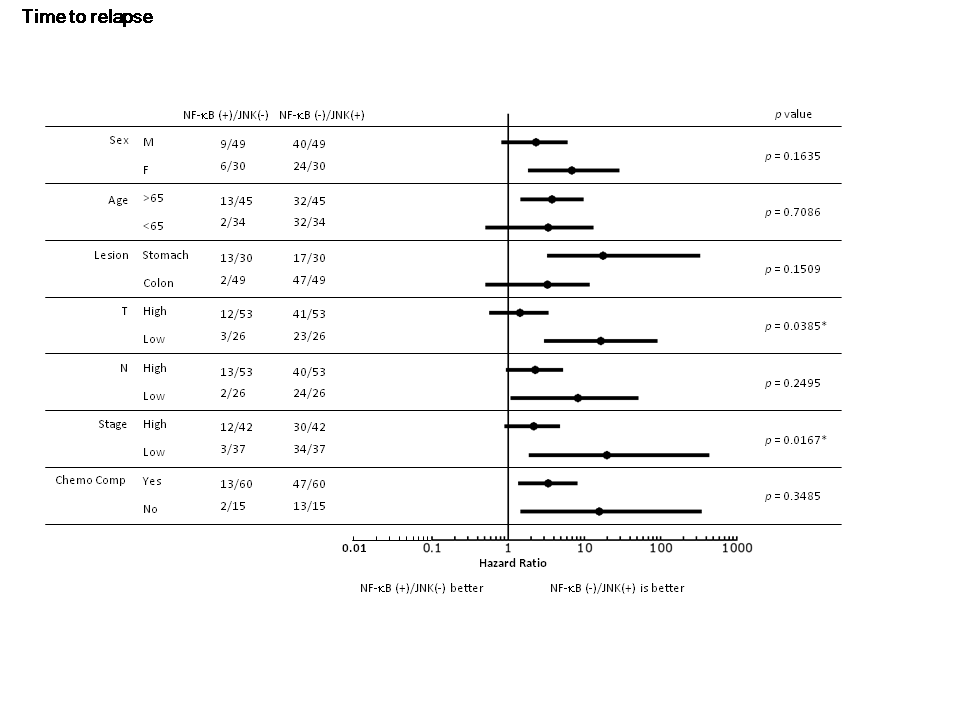

Supplement: Figure S8 — Hazard ratio for relapse and p values for the interaction of NF-κB/JNK status and clinical subgroup categories. (TIF) [file pone.0043236.s008.tif]

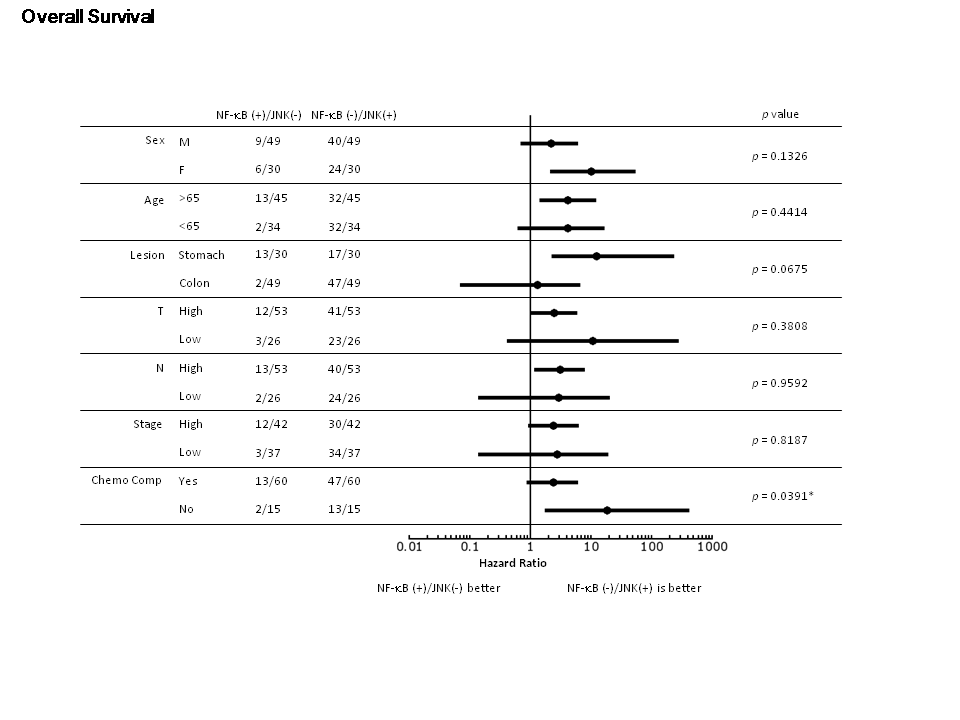

Supplement: Figure S9 — Hazard ratio for death and p values for the interaction of NF-κB/JNK status and clinical subgroup categories. (TIF) [file pone.0043236.s009.tif]

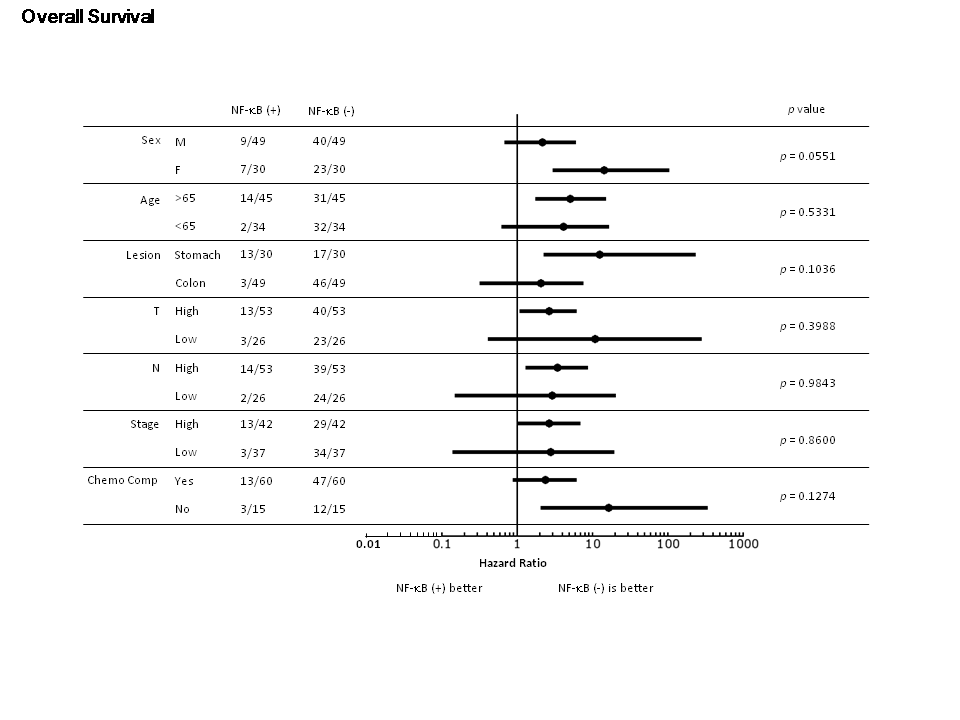

Supplement: Figure S10 — Hazard ratio for death and p values for the interaction of NF-κB status and clinical subgroup categories. (TIF) [file pone.0043236.s010.tif]

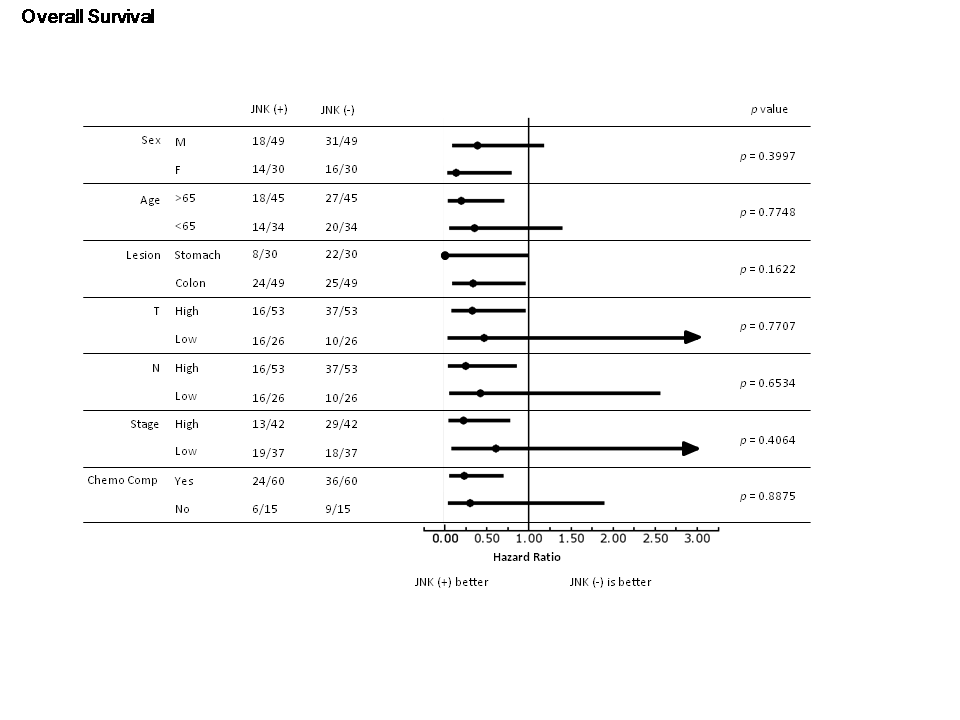

Supplement: Figure S11 — Hazard ratio for death and p values for the interaction of JNK status and clinical subgroup categories. (TIF) [file pone.0043236.s011.tif]

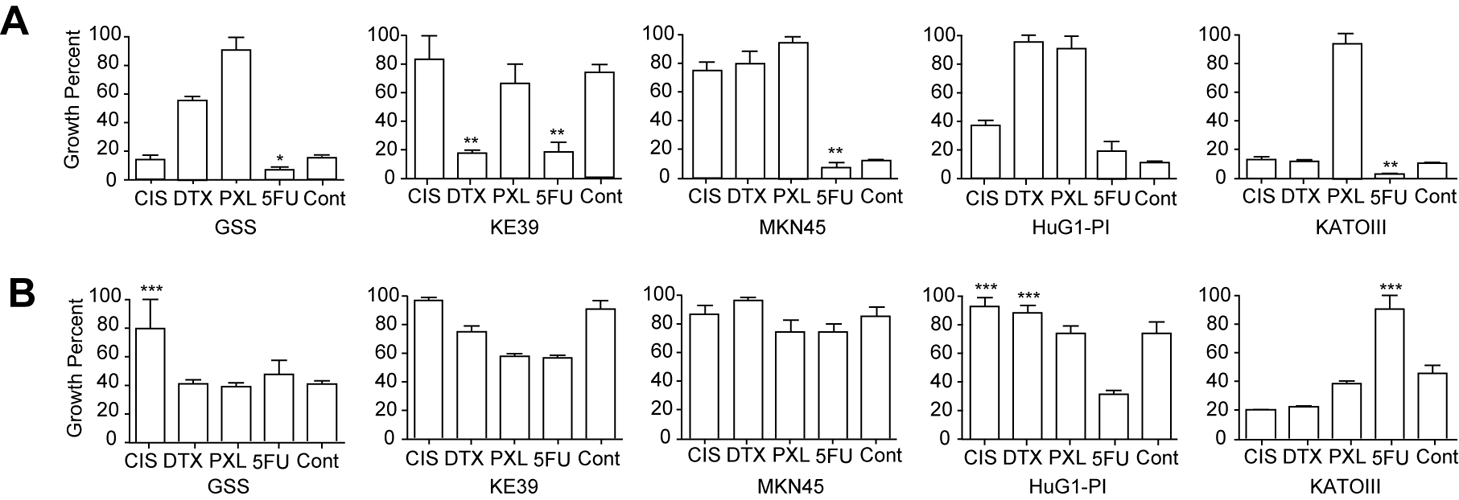

Supplement: Figure S12 — Enhanced growth inhibitory effect by p65 gene knock down. Growth inhibitory effect of anticancer drugs at a concentration that elicits a 50% growth inhibitory (GI50) effect after 48 h of incubation in gastric cancer cell lines after transfection of siRNA for NF-κB p65 subunit (A) and JNK (B). (TIF) [file pone.0043236.s012.tif]
