# Supplementary material for: Molecular Marker Identification for Relapse Prediction in 5-FU-Based Adjuvant Chemotherapy in Gastric and Colorectal Cancers
Source: PLoS One. 2012 Aug 14;7(8):e43236. doi: 10.1371/journal.pone.0043236 (PMC3419205; doi:10.1371/journal.pone.0043236)
Supplement: Table S1 — Candidate Markers Identified From Quantitative Protein Expression Analysis and Chemosensitivity Assay (DOC) [file pone.0043236.s013.doc]

| **Table S1.** Candidate Markers Identified From Quantitative Protein Expression Analysis and Chemosensitivity Assay | | |
| --- | --- | --- |
| **Candidate Markers** | ***r**** | **95% CI** |
| JNK | 0.83 | 0.47 - 0.96 |
| GSK-3β | 0.76 | 0.30 - 0.93 |
| elF4E | 0.67 | 0.12 - 0.92 |
| Caspase-7 | 0.66 | 0.10 - 0.90 |
| NFκB p65 | 0.60 | 0.01 - 0.88 |
| Keratin 8 | -0.71 | -0.92 - 0.20 |
| Keratin 17 | -0.73 | -0.93 - -0.23 |
| Keratin 18 | -0.75 | -0.93 - -0.28 |
| Abbreviations: JNK, c-JUN N-terminal kinases; GSK-3β, Glycogen Synthase Kinase 3β; eIF4E, Eukaryotic Initiation Factor 4E; NF-κB p65, Nuclear factor-κB p65. *Correlation coefficient was calculated from an AP matrix generated from A (drug x cell) and P (cell x protein) matrices. | | |
