# Supplementary material for: Molecular Marker Identification for Relapse Prediction in 5-FU-Based Adjuvant Chemotherapy in Gastric and Colorectal Cancers
Source: PLoS One. 2012 Aug 14;7(8):e43236. doi: 10.1371/journal.pone.0043236 (PMC3419205; doi:10.1371/journal.pone.0043236)
Supplement: Table S2 — Primary antibodies Used for Candidate Marker Validation on TMAs (DOC) [file pone.0043236.s014.doc]

| **Table S2.** Antibodies Used for Candidate Marker Validation on TMAs | | | |
| --- | --- | --- | --- |
| **Antibody Name** | **Vendor** | **Catalogue Number** | **Dilution** |
| NF-κB p65 (C22B4) Rabbit mAB | CST | 4764 | 1:1000 |
| pan-JNK/SAPK1 | BD | 610627 | 1:250 |
| Keratin 8 Ab-4 (Clone TS1) | TFS | MS-997 | 1:200 |
| Keratin 17 Ab-1 | TFS | MS-489 | 1:40 |
| Keratin 18 Ab-1 (Clone DC10) | TFS | MS-142 | 1:100 |
| Caspase-7/MCH-3 | BD | 610812 | 1:500 |
| eIF4E (C46H6) Rabbit mAB | CST | 2067 | 1:1000 |
| GSK-3β (27C10) Rabbit mAB | CST | 9315 | 1:1000 |
| *TS (TS106) | SC | sc-33679 | 1:100 |
| p53 Ab-6 (Clone DO-1) | TFS | MS-187 | 1:200 |
| Monoclonal Anti-P-Glycoprotein (**MDR) Clone4 | SA | P7965 | 1:1000 |
| Seven antibodies from the top are listed from the in vitro chemosensitivity marker identification demonstrated in Fig. 1. Antibodies for TS, p53, and MDR are of general interest. Abbreviation: *TS, Thymidylate Synthase; **MDR, Multi Drug Resistant; CST, Cell Signaling Technology; BD, BD Biosciences; TFS, Thermo Fisher Scientific; SC, Santa Cruz Biotechnology; SA, Sigma-Aldrich | | | |
