# Supplementary material for: Molecular Marker Identification for Relapse Prediction in 5-FU-Based Adjuvant Chemotherapy in Gastric and Colorectal Cancers
Source: PLoS One. 2012 Aug 14;7(8):e43236. doi: 10.1371/journal.pone.0043236 (PMC3419205; doi:10.1371/journal.pone.0043236)
Supplement: Table S3 — Clinicopathological Features of the State of Relapse (DOC) [file pone.0043236.s015.doc]

| **Table S3.** Clinicopathological Features of the State of Relapse | | | | | |
| --- | --- | --- | --- | --- | --- |
| **Variable** | **Relapse (+)** | | **Relapse (-)** | | ***p**** |
|  | **No.** | **%** | **No.** | **%** |  |
| Stomach (n = 30) |  |  |  |  |  |
| Age |  |  |  |  |  |
| ≥60 | 8 | 26.7 | 13 | 43.3 | 0.675 |
| <60 | 2 | 6.7 | 7 | 23.3 |  |
| Sex |  |  |  |  |  |
| Male | 7 | 23.3 | 12 | 40.0 | 0.702 |
| Female | 3 | 10.0 | 8 | 26.7 |  |
| T factor |  |  |  |  |  |
| 2 | 2 | 6.7 | 12 | 40.0 | 0.109 |
| 3 | 8 | 26.7 | 8 | 26.7 |  |
| N factor |  |  |  |  |  |
| 0 | 1 | 3.3 | 1 | 3.3 | 0.187 |
| 1 | 3 | 10.0 | 17 | 56.7 |  |
| 2 | 5 | 16.7 | 2 | 6.7 |  |
| 3 | 1 | 3.3 | 0 | 0 |  |
| Stage (stomach) |  |  |  |  |  |
| II | 2 | 6.7 | 11 | 36.7 | 0.0065 |
| IIIA | 3 | 10.0 | 9 | 30.0 |  |
| IIIB | 4 | 13.3 | 0 | 0.0 |  |
| IV | 1 | 3.3 | 0 | 0.0 |  |
| Chemotherapy |  |  |  |  |  |
| Completed | 7 | 23.3 | 18 | 60.0 | 0.300 |
| Suspended | 3 | 10.0 | 2 | 6.7 |  |
| Time to relapse (Years) |  |  |  |  |  |
| Median | 3.02 | | | |  |
| Mean | 3.23 | | | |  |
| Range | 0.90 - 7.00 | | | |  |
|  |  |  |  |  |  |
| Colon (n =49) |  |  |  |  |  |
| Age |  |  |  |  |  |
| ≥60 | 18 | 36.7 | 20 | 40.8 | 0.162 |
| <60 | 2 | 4.1 | 9 | 18.4 |  |
| Sex |  |  |  |  |  |
| Male | 14 | 28.6 | 16 | 32.7 | 0.377 |
| Female | 6 | 12.2 | 13 | 26.5 |  |
| T factor |  |  |  |  |  |
| 2 | 1 | 2.0 | 11 | 22.4 | 0.012 |
| 3 | 15 | 30.6 | 12 | 24.5 |  |
| 4 | 4 | 8.2 | 6 | 12.2 |  |
| N factor |  |  |  |  |  |
| 0 | 5 | 10.2 | 19 | 38.8 | 0.119 |
| 1 | 14 | 28.6 | 10 | 20.4 |  |
| 2 | 1 | 2.0 | 0 | 0 |  |
| Stage (colorectal) |  |  |  |  |  |
| I | 1 | 2.0 | 7 | 14.3 | 0.0037 |
| IIa | 4 | 8.2 | 7 | 14.3 |  |
| IIb | 0 | 0.0 | 5 | 10.2 |  |
| IIIa | 0 | 0.0 | 4 | 8.2 |  |
| IIIb | 14 | 28.6 | 6 | 12.2 |  |
| IIIc | 1 | 2.0 | 0 | 0.0 |  |
| Chemotherapy |  |  |  |  |  |
| Completed | 14 | 28.6 | 21 | 42.9 | NA |
| Suspended | 5 | 10.2 | 5 | 10.2 |  |
| Unknown | 1 | 2.0 | 3 | 6.1 |  |
| Time to relapse (Years) |  |  |  |  |  |
| Median | 1.56 | | | |  |
| Mean | 1.65 | | | |  |
| Range | 0.72 - 3.33 | | | |  |
| NOTE. Abbreviations: NA, not applicable. Chemotherapy completed, continued chemotherapy for 0.5 years for colorectal and 1 year for stomach.*Age, Sex, and Chemotherapy were tested with a Fisher’s exact test; all others were tested with a χ^2^ test. | | | | | |
