# Supplementary material for: Molecular Marker Identification for Relapse Prediction in 5-FU-Based Adjuvant Chemotherapy in Gastric and Colorectal Cancers
Source: PLoS One. 2012 Aug 14;7(8):e43236. doi: 10.1371/journal.pone.0043236 (PMC3419205; doi:10.1371/journal.pone.0043236)
Supplement: Table S4 — Clinicopathological Features of Immunohistochemical Status (DOC) [file pone.0043236.s016.doc]

| **Table S4.** Clinicopathological Features of Immunohistochemical Status | | | | | | | | | | | | |
| --- | --- | --- | --- | --- | --- | --- | --- | --- | --- | --- | --- | --- |
| **Variable** | **NF-κB** | | | | | | **JNK** | | | | | |
|  | **Positive** | | **Negative** | | | ***p**** | **Positive** | | | **Negative** | | ***p**** |
|  | **No.** | **%** | **No.** | | **%** |  | **No.** | **%** | | **No.** | **%** |  |
| Stomach (n = 30) |  |  |  | |  |  |  |  | |  |  |  |
| Age |  |  |  | |  |  |  |  | |  |  |  |
| >60 | 12 | 40.0 | 9 | | 30.0 | 0.042 | 7 | 23.3 | | 14 | 46.7 | 0.374 |
| ≤60 | 1 | 3.. | 8 | | 26.7 |  | 1 | 3.3 | | 8 | 26.7 |  |
| Sex |  |  |  | |  |  |  |  | |  |  |  |
| Male | 9 | 30.0 | 10 | | 33.3 | 0.708 | 5 | 16.7 | | 14 | 46.7 | 1.00 |
| Female | 4 | 13.3 | 7 | | 23.3 |  | 3 | 10.0 | | 8 | 26.7 |  |
| T factor |  |  |  | |  |  |  |  | |  |  |  |
| 2 | 3 | 10.0 | 11 | | 36.7 | 0.021 | 4 | 13.3 | | 10 | 33.3 | 0.826 |
| 3 | 10 | 33.3 | 6 | | 20.0 |  | 4 | 13.3 | | 12 | 40.0 |  |
| N factor |  |  |  | |  |  |  |  | |  |  |  |
| 0 | 1 | 3.3 | 1 | | 3.3 | 0.140 | 1 | 3.3 | | 1 | 3.3 | 0.605 |
| 1 | 6 | 20.0 | 14 | | 46.7 |  | 6 | 20.0 | | 14 | 46.7 |  |
| 2 | 5 | 16.7 | 2 | | 6.7 |  | 1 | 3.3 | | 6 | 20.0 |  |
| 3 | 1 | 3.3 | 0 | | 0 |  | 0 | 0 | | 1 | 3.3 |  |
| Stage |  |  |  | |  |  |  |  | |  |  |  |
| II | 2 | 6.7 | 11 | | 36.7 | .04 | 4 | 13.3 | | 9 | 30.0 | .53 |
| IIIA | 7 | 23.3 | 5 | | 16.7 |  | 4 | 13.3 | | 8 | 26.7 |  |
| IIIB | 3 | 10.0 | 1 | | 3.3 |  | 0 | 0.0 | | 4 | 13.3 |  |
| IV | 1 | 3.3 | 0 | | 0.0 |  | 0 | 0.0 | | 1 | 3.3 |  |
| Histology |  |  |  | |  |  |  |  | |  |  |  |
| Intestinal | 6 | 20.0 | 7 | | 23.3 | 1.00 | 3 | 10.0 | | 10 | 33.3 | 1.00 |
| Diffuse | 7 | 23.3 | 10 | | 33.3 |  | 5 | 16.7 | | 12 | 40.0 |  |
| Lymphatic Invasion |  |  |  | |  |  |  |  | |  |  |  |
| 1 | 4 | 13.3 | 14 | | 46.7 | 0.100 | 6 | 20.0 | | 12 | 40.0 | 0.272 |
| 2 | 8 | 26.7 | 2 | | 6.7 |  | 1 | 3.3 | | 9 | 30.0 |  |
| 3 | 1 | 3.3 | 1 | | 3.3 |  | 1 | 3.3 | | 1 | 3.3 |  |
| Venous Invasion |  |  |  | |  |  |  |  | |  |  |  |
| 0 | 0 | 0 | 2 | | 6.7 | 0.114 | 0 | 0 | | 2 | 6.7 | 0.509 |
| 1 | 6 | 20.0 | 12 | | 40.0 |  | 6 | 20.0 | | 12 | 40.0 |  |
| 2 | 5 | 16.7 | 2 | | 6.7 |  | 1 | 3.3 | | 6 | 20.0 |  |
| 3 | 2 | 6.7 | 1 | | 3.3 |  | 1 | 3.3 | | 2 | 6.7 |  |
| Chemotherapy |  |  |  | |  |  |  |  | |  |  |  |
| Completed | 11 | 36.7 | 14 | | 46.7 | 1.00 | 7 | 23.3 | | 18 | 60.0 | 1.00 |
| Suspended | 2 | 6.7 | 3 | | 10.0 |  | 1 | 3.3 | | 4 | 13.3 |  |
| Relapse |  |  |  | |  |  |  |  | |  |  |  |
| Absent | 4 | 13.3 | 16 | | 53.3 | 0.0004 | 8 | 26.7 | | 12 | 40.0 | 0.029 |
| Present | 9 | 30.0 | 1 | | 3.3 |  | 0 | 0 | | 10 | 33.3 |  |
| Time to Relapse (Years) |  |  |  | |  |  |  |  | |  |  |  |
| Median | 1.81 | | | NA* | |  | NA | | | 1.56 | |  |
| Mean | 1.70 | | | NA | |  | NA | | | 1.65 | |  |
| Range | 0.72 - 3.33 | | | NA | |  | NA | | | 0.72 - 3.34 | |  |
|  |  |  |  | |  |  |  | |  |  |  |  |
| Colon (n = 49) |  |  |  | |  |  |  | |  |  |  |  |
| Age |  |  |  | |  |  |  | |  |  |  |  |
| >60 | 3 | 6.1 | 35 | | 71.4 | 1.00 | 16 | | 32.7 | 22 | 44.9 | 0.096 |
| ≤60 | 0 | 0 | 11 | | 22.4 |  | 8 | | 16.3 | 3 | 6.1 |  |
| Sex |  |  |  | |  |  |  | |  |  |  |  |
| Male | 0 | 0 | 30 | | 6.1 | 0.053 | 13 | | 26.5 | 17 | 34.7 | 0.387 |
| Female | 3 | 6.1 | 16 | | 3.3 |  | 11 | | 22.4 | 8 | 16.3 |  |
| T factor |  |  |  | |  |  |  | |  |  |  |  |
| 2 | 0 | 0 | 12 | | 24.5 | 0.155 | 12 | | 24.5 | 0 | 0 | <0.0001 |
| 3 | 3 | 6.1 | 24 | | 49.0 |  | 8 | | 16.3 | 19 | 38.8 |  |
| 4 | 0 | 0 | 10 | | 20.4 |  | 4 | | 8.2 | 6 | 12.2 |  |
| N factor |  |  |  | |  |  |  | |  |  |  |  |
| 0 | 1 | 2.0 | 23 | | 46.9 | 0.783 | 15 | | 30.6 | 9 | 18.4 | 0.111 |
| 1 | 2 | 4.1 | 22 | | 44.9 |  | 9 | | 18.4 | 15 | 30.6 |  |
| 2 | 0 | 0 | 1 | | 20.4 |  | 0 | | 0 | 1 | 2.0 |  |
| Stage (colorectal) |  |  |  | |  |  |  | |  |  |  |  |
| I** | 0 | 0.0 | 8 | | 16.3 | 0.86 | 8 | | 16.3 | 0 | 0.0 | 0.0005 |
| IIa | 1 | 2.0 | 10 | | 20.4 |  | 3 | | 6.1 | 8 | 16.3 |  |
| IIb | 0 | 0.0 | 5 | | 10.2 |  | 4 | | 8.2 | 1 | 2.0 |  |
| IIIa | 0 | 0.0 | 4 | | 8.2 |  | 4 | | 8.2 | 0 | 0.0 |  |
| IIIb | 2 | 4.1 | 18 | | 36.7 |  | 5 | | 10.2 | 15 | 30.6 |  |
| IIIc | 0 |  | 1 | | 2.0 |  | 0 | | 0.0 | 1 | 2.0 |  |
| Lymphatic Invasion |  |  |  | |  |  |  | |  |  |  |  |
| 0 | 0 | 0 | 2 | | 4.1 | 0.733 | 1 | | 2.0 | 1 | 2.0 | 0.956 |
| 1 | 2 | 4.1 | 36 | | 73.5 |  | 19 | | 38.8 | 19 | 38.8 |  |
| 2 | 1 | 2.1 | 8 | | 16.3 |  | 4 | | 8.2 | 5 | 10.2 |  |
| Venous Invasion |  |  |  | |  |  |  | |  |  |  |  |
| 1 | 2 | 4.1 | 35 | | 71.4 | 0.775 | 20 | | 40.8 | 17 | 34.7 | 0.393 |
| 2 | 1 | 2.0 | 9 | | 18.4 |  | 3 | | 6.1 | 7 | 14.3 |  |
| 3 | 0 | 0 | 2 | | 4.1 |  | 1 | | 1 | 1 | 2.0 |  |
| Chemotherapy |  |  |  | |  |  |  | |  |  |  |  |
| Completed | 1 | 2.0 | 9 | | 18.4 | 0.692 | 5 | | 10.2 | 5 | 10.2 | 0.996 |
| Suspended | 2 | 4.1 | 33 | | 67.3 |  | 17 | | 34.7 | 18 | 36.7 |  |
| Unknown | 0 | 0 | 4 | | 8.2 |  | 2 | | 4.1 | 2 | 4.1 |  |
| Relapse |  |  |  | |  |  |  | |  |  |  |  |
| Absent | 0 | 0 | 29 | | 59.2 | 0.062 | 19 | | 38.8 | 10 | 20.4 | 0.009 |
| Present | 3 | 6.1 | 17 | | 34.7 |  | 5 | | 10.2 | 15 | 30.6 |  |
| Time to Relapse (Years) |  |  |  | |  |  |  | |  |  |  |  |
| Median | 1.52 | | 1.30 | | |  | 1.18 | | | 1.67 | |  |
| Mean | 2.20 | | 1.71 | | |  | 1.03 | | | 2.03 | |  |
| Range | 1.52 - 3.57 | | 0.267 - 4.47 | | |  | 0.47 - 1.52 | | | 0.27 - 4.47 | |  |
| *Age, Sex, Histology, and Relapse were tested with a Fisher’s exact test (2-tail); T and N factors, Stage, Lymphatic Invasion, Venous Invasion, and Chemotherapy, with a χ^2^ test; Time to Relapse, with a *t* test. **The depth of all tumors reached to muscularis propria. | | | | | | | | | | | | |
